# The time-frequency analysis of the acoustic signal produced in underwater discharges based on Variational Mode Decomposition and Hilbert–Huang Transform

**DOI:** 10.1038/s41598-022-27359-5

**Published:** 2023-01-02

**Authors:** Zhen Han, Xiaobing Zhang, Bing Yan, Liang Qiao, Zhigang Wang

**Affiliations:** 1 College of Weapons Engineering, Naval University of Engineering, Wuhan, 430033 China; 2 School of Electrical and Electronic Engineering, Baoji University of Arts and Sciences, Baoji, 721016 China; 3 Department of Weapons, Naval Petty Officer Academy, Bengbu, 233012 China

**Keywords:** Plasma physics, Acoustics, Electrical and electronic engineering, Engineering, Physics

## Abstract

The experiments of underwater discharges in an anechoic pool were carried out and analysis of the time-frequency characteristics of the acoustic signals was conducted based on Variational Mode Decomposition and Hilbert–Huang Transform (VMD-HHT). We propose a relative center frequency difference method to determine the decomposition numbers *K* which has to be given before the application of VMD and the result is satisfying. The HHT spectrum and marginal spectrum are obtained, then, some valuable conclusions are drawn. The high-frequency components of the acoustic signal are mainly attributed to the shock wave, and the low-frequency components mostly result from the bubble pulse. The frequency range of the acoustic signal is basically from 0 to 90kHz, and the ratio of energy in the low-frequency band(0–4kHz) to that of the total acoustic signal is up to 55.56%. Furthermore, this ratio versus gaps is also explored and it has the minimum at the gap of 1.5 mm which is the optimal gap for the peak pressure and radiated energy of the acoustic signal. Therefore, we can not obtain the maximum energy of the acoustic signal and the maximum ratio in the low-frequency band simultaneously.

## Introduction

Strong acoustic signals which are extensively applied in marine exploration, underwater communication, target detection, water treatment, and other fields can be induced by explosions^1^, airguns^2^, transducers^3^, laser^4–7^, underwater discharges^8,9^, and so on. This paper focus on the acoustic signals originating from underwater discharges. High electric field acts on the electrodes immersed in the liquid and caused the stored electric energy to be instantaneously released into the discharge channel formed between the electrodes, which brings about the high-temperature and high-pressure plasma together with optical emission^10^, active species^11^ and thermal diffusion^12,13^. When the plasma channel expands outward, the shock wave comes into being. For the better application of this acoustic signal, obtaining the accurate characteristic of acoustic signals produced in underwater discharges, especially the distribution of time and frequency, is of good significance. Some researchers^14^ give amplitude-frequency characteristics of acoustic signals by FFT. Nevertheless, FFT, together with some time-frequency analysis methods based on Fourier transform, such as short-time Fourier transform (STFT), Gabor transform, and Wigner–Ville distribution, is suitable for processing linear and stationary signals. As for nonstationary signals, they are unable to supply the exact frequency-domain characteristic. Thus, suitable signal processing methods, such as Wavelet and HHT, should be taken into consideration.

Wavelet^15^ is a powerful tool for analyzing transient and nonstationary signals. Unfortunately, the wavelet base function must be selected manually and can not be changed during signal processing. If the wavelet base function is not appropriate, the analysis result is not satisfying. Compared to wavelet analysis, HHT has good adaptability, which means there is no need to choose a base function for decomposing signals in advance. As a new and valid method in nonstationary signals processing, HHT which was proposed by Huang in 1998^16^, is widely used to analyze seismic signals^17^, ECG signals^18^, underwater explosive explosion signals^19^, and so on. The acoustic signal generated from underwater discharges is also transient and nonstationary just like the one generated by underwater explosions. Thus, HHT is employed in this paper. The key of HHT lies in the signal decomposition method, which is mostly done by Empirical mode decomposition (EMD)^20^. Liang Qiao^21^ gives the time-frequency spectrums of acoustic signals produced by underwater discharges based on HHT. However, a severe disadvantage of EMD is mode mixing, which was firstly found by Huang during the decomposition of discontinuous signals. Specifically speaking, the same characteristic time scale exists in multiple IMFs simultaneously, or multiple characteristic time scales live in one IMF. Mode mixing leads to the disability of IMFs from representing an actual physical process, which makes nonsense for the HHT spectrum. Therefore, eliminating mode mixing validly is of great importance.

VMD with a characteristic of suppressing mode fixing is selected in this paper. However, the determination of decomposition numbers *K* before the usage of VMD is a challenge. We propose a relative center frequency difference method to get this job done and make a comparison with the energy difference method. The validity is verified, and the result is satisfying.

## Experimental apparatus

**Figure 1:**
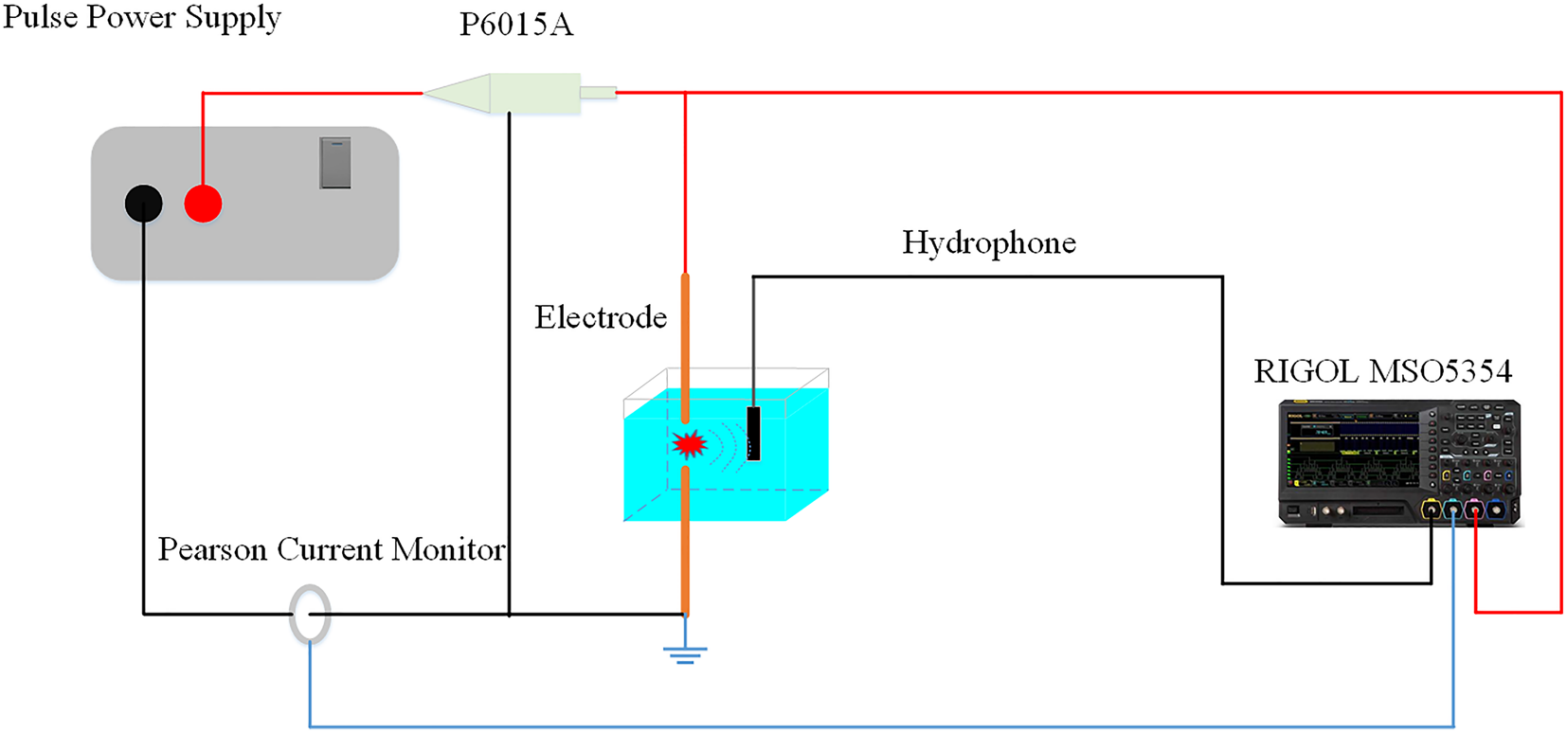
Experimental apparatus of the underwater discharge system.

A large number of underwater discharge experiments were conducted in an anechoic water pool. The conductivity of the water is 0.35 mS/cm and the water temperature is 26.2 °C. Stainless steel rod-to-rod electrodes with a length of 150mm and a diameter of 5mm were adopted in our experiments. The center of the electrode gap is 1 m underwater, and the electrode gap distance is adjustable from 0.5 mm to 1 cm. The pulse power supply with a charge voltage of 10 kV and an energy storage capacitance of 0.11 μF is operated through a manual trigger switch. A high-voltage probe (Tektronix P6015A) monitors the voltage across the electrode gap. A Rogowski coil (Pearson 2879) is sheathed on the transmission line to measure the current through the circuit. A hydrophone with a sensitivity level of − 205 dB re 1V/μPa in the range of 5 Hz–15 MHz was placed 1 m underwater and 1 m away from the center of the stainless steel electrode gap to receive acoustic signals produced by underwater discharges and to transform them into voltage signals. A digital storage oscilloscope (RIGOL MSO5354) was selected to store and display all concerning signals. A diagram of the experimental apparatus was shown in Fig. 1.

## Results and discussion

### Time-domain characteristics of acoustic signals produced by a spark discharge

**Figure 2:**
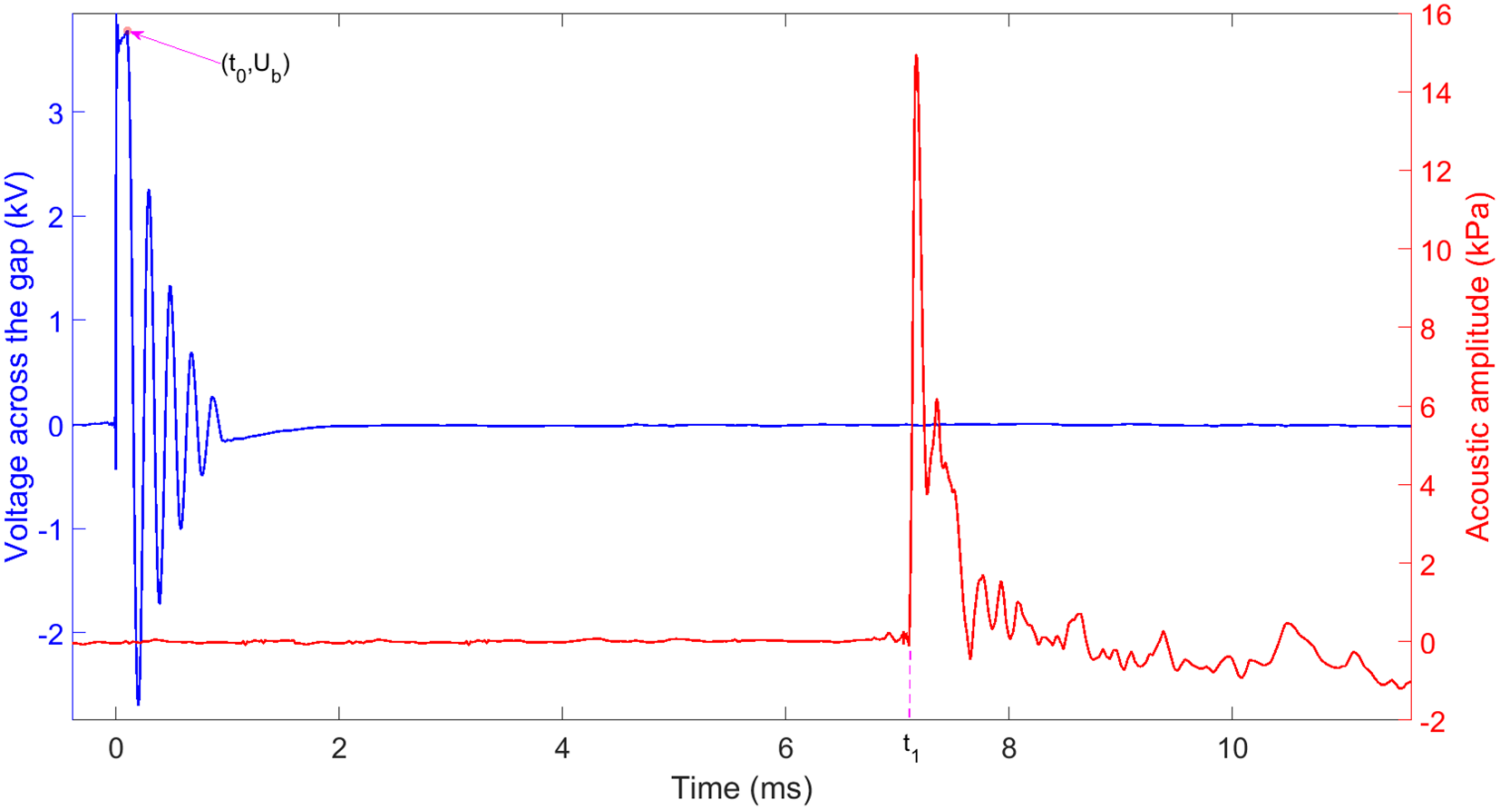
A typical waveform of voltage across the electrode gap of 0.5 mm as well as a subsequent acoustic waveform.

The receiving sensitivity of a hydrophone is $M=\frac{u}{p}$, where *u* is the output voltage signal of the hydrophone with a unit of V and *p* is the received acoustic signal by the hydrophone with a unit of μPa. Then the sensitivity level of the hydrophone can be expressed as $SL=20\lg \frac{M}{M_0}$, where $M_0=1\,V/\upmu$Pa is the reference sensitivity. In our experiments, the sensitivity level $SL=-\,205\,dB$. Thus, the acoustic pressure *p* is computed by $p=\frac{u}{M_0}10^{-\frac{SL}{20}}$ with a unit of μPa. A typical waveform of voltage across the electrode gap of 0.5 mm and a subsequent acoustic signal is indicated in Fig. 2.

**Figure 3:**
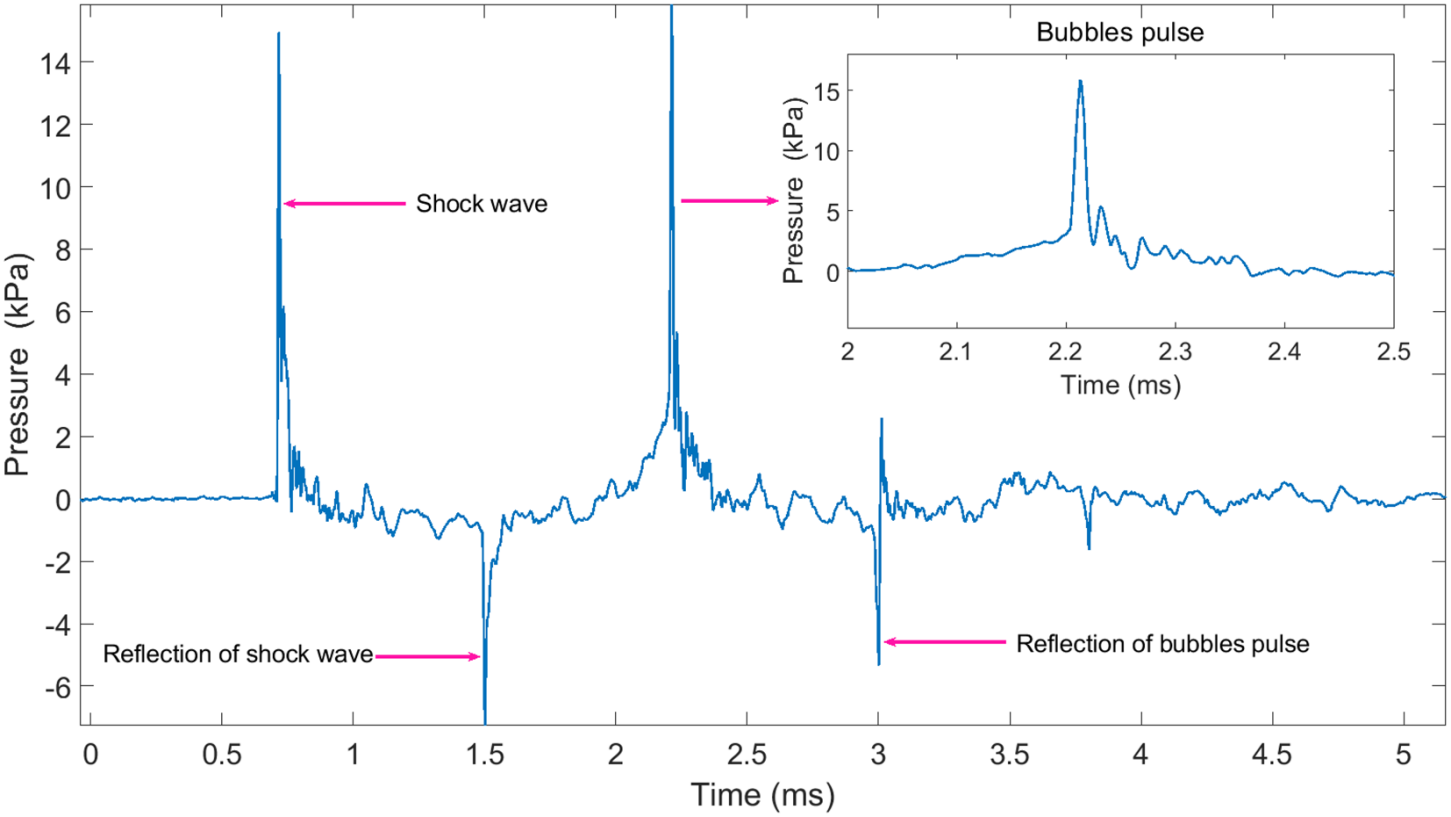
The total acoustic signal produced in a spark discharge.

When $t=0$, the trigger switch is closed, and the charge voltage is applied across the electrode gap. From 0 to $t_0$, streamers appear at the anode of the electrode and propagate to the cathode under the function of the applied electric field. This period during which the plasma channel is formed is called the pre-breakdown phase of a spark discharge. The electrode gap is broken down at time $t_0$, and then the voltage across the gap drops violently due to the extremely low resistance of the plasma channel, which is full of high-temperature and high-pressure plasma. The voltage across the gap oscillates several cycles after time $t_0$ and drops to 0 eventually with the deposited electrical energy into the channel consumed. Almost at $t_0$, the plasma channel expands rapidly and compresses the surrounding water, which leads to the generation of the shock wave. The shock wave is seen at time $t_1$ in Fig. 2 because the hydrophone is 1 m away from the electrodes center. The formation mechanism of the shock wave can be explained by the piston model^22^. The front of the shock wave is considerably steep, with a rising rate being 2.15 kPa/μs. After reaching the peak (14.9 kPa), the amplitude of the shock wave decreases approximately according to the exponential law. As is seen, the shock wave has a short pulse width of about 52.8 μs. Next, we briefly describe the formation process of the bubble pulse. The plasma channel expands due to the internal pressure being more considerable than the static pressure of water. After the deposited electrical energy is consumed, the channel can be regarded as a bubble. With increase of the bubble volume, the internal pressure declines. When the internal pressure is equal to the static pressure, the bubble continue to expand because of inertia. As it reaches the maximum radius, the bubble ceases to expand and contract inversely. Similarly, the bubble stops contracting until the radius reaches the minimum. At this moment, the pressure inside the bubble gets the peak again. This is the first bubble pulse. Repeating this process can form a second bubble pulse or even a third bubble pulse. Finally, the bubble collapses due to energy depletion. The total acoustic signal containing the shock wave (also called the expansion pulse) and the bubble pulse (also called the collapse pulse) is shown in Fig. 3.

**Figure 4:**
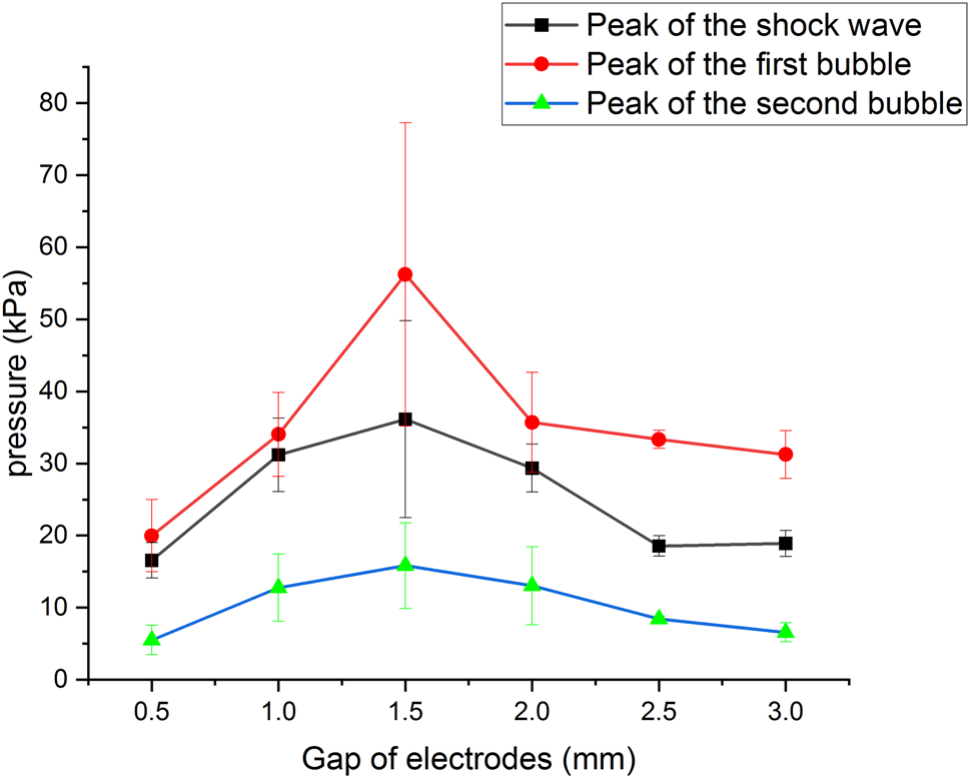
The peak pressure versus gaps.

Compared to the shock wave, the rising edge of the bubble pulse is not that steep and the peak pressure (15.85 kPa) is more significant. The width of the bubble pulse is 500 μs, which is nearly ten times bigger than that of the shock wave. Between the shock wave and the bubble pulse, a water surface reflection signal of the shock wave with a negative amplitude is visible in Fig. 3. After the bubble pulse, there is also a corresponding water surface reflection signal. In some data sets, more bubble pulses and corresponding water surface reflection signals are observed. Underwater discharge experiments were carried out with different gaps, including 0.5 mm, 1 mm, 1.5 mm, 2 mm, 2.5 mm, and 3 mm. Three discharges were performed at each gap. The error bars were plotted to show the statistical results. Each data point represents the mean value and the error adopts the standard deviation. The peak pressure versus gaps is shown in Fig. 4.

**Figure 5:**
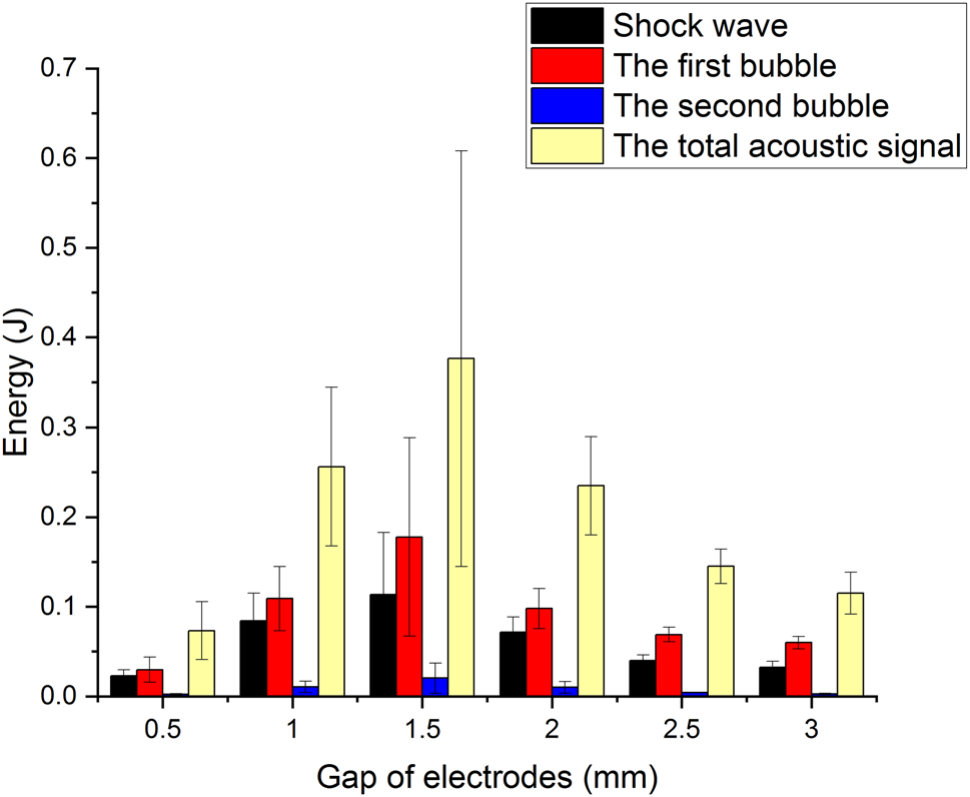
The energy versus gaps.

For a fixed gap, the first bubble pulse has the maximum peak pressure while the second bubble pulse has the minimum one. At a gap of 1.5 mm, the peak pressure of the shock wave, the first bubble pulse, and the second bubble pulse all achieve the maximum. Therefore, 1.5 mm is called the optimal gap. The energy of a shock wave can be expressed as $E_{sh}=\frac{4\pi s^2}{\rho _0c_0} \int _{0}^{\tau _{sh}} {p^2_{sh}}dt$ with the unit of J, where $\rho _0$ is the density of water, $c_0$ is the sound speed in the water, which is thought as the speed of the shock wave approximately, *s* is the distance between the center of the electrode and the hydrophone and $\tau _{sh}$ is the pulse width of the shock wave. Correspondingly, the radiated energy of the first bubble pulse can be expressed as $E_{FB}=\frac{4\pi s^2}{\rho _0c_0} \int _{0}^{\tau _{FB}} {p^2_{FB}}dt$, where $\tau _{FB}$ is the width of the first bubble pulse, and the radiated energy of the second bubble pulse can be expressed as $E_{SB}=\frac{4\pi s^2}{\rho _0c_0} \int _{0}^{\tau _{SB}} {p^2_{SB}}dt$ , where $\tau _{SB}$ is the width of the second bubble pulse. The total energy of the acoustic signal is the sum of the energy of the shock wave, the first bubble pulse, the second bubble pulse, and the reflection signals. According to the calculation methods mentioned above, the energy versus gaps is obtained and shown in Fig. 5.

**Figure 6:**
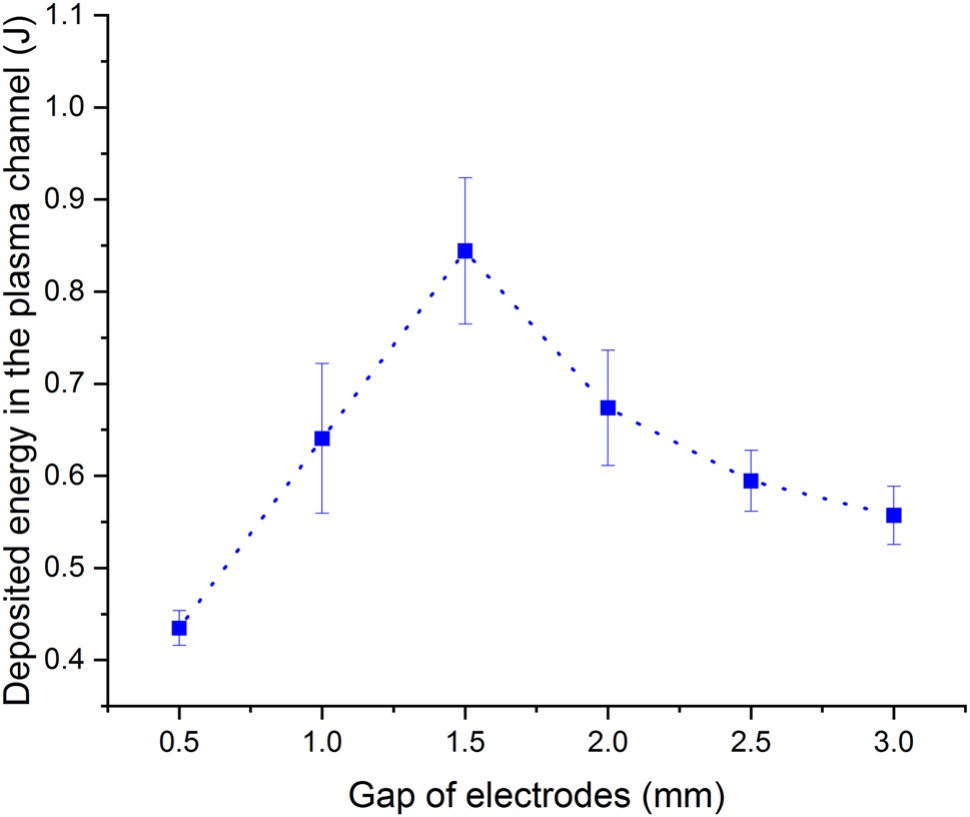
The deposited electrical energy versus gaps.

Obviously, the optimal gap of 1.5 mm also exists. From the energy balance point of view, the energy of the acoustic signal originates from the deposited electrical energy into the plasma channel. Therefore, the relationship between the deposited electrical energy and gaps needs to be explored. The electrical energy deposited into the plasma gap is computed by $E_{ch}=\int {i^2R_{ch}}dt$, where *i* is the measured current through the gap and $R_{ch}$ is the constant resistance of the channel which is determined by the difference of the total resistance of the circuit minus the external circuit resistance. The external circuit resistance can be obtained by short-circuit method and the total resistance of the circuit is usually obtained by the fitness to measured current on the basis of analytic expression for current coming from the RLC equivalent circuit model^23^. The deposited electrical energy versus gaps is shown in Fig. 6.

The changing trend of the deposited electrical energy is just like the one of the peak pressure and the radiated energy shown in Figs. 4 and 5. At the gap of 1.5 mm, the deposited electrical energy reaches the maximum. This is the reason why the optimal gap of 1.5 mm can be seen in Figs. 4 and 5. A more clear explanation of the optimal gap is made as follows. The radiated energy of the acoustic signal depends on the electrical energy deposited into the plasma channel, which is affected by the remained electrical energy of the capacitor at the break-down moment and the channel resistance^24^. In the case of short gaps, although the remained electrical energy is significant due to the short pre-breakdown period, the low channel resistance compared to the external circuit resistance results in a small amount of deposition energy in the plasma channel. As for long gaps, the channel resistance is higher than the external circuit resistance, but the remained electrical energy is small because of the long pre-breakdown period, which also brings about a small amount of deposition energy. The plasma channel can be regarded as a load in the equivalent RLC circuit. Then, based on impedance matching theory, When the channel resistance is as close as the external circuit resistance, the channel resistance gets the maximum power and energy.

**Figure 7:**
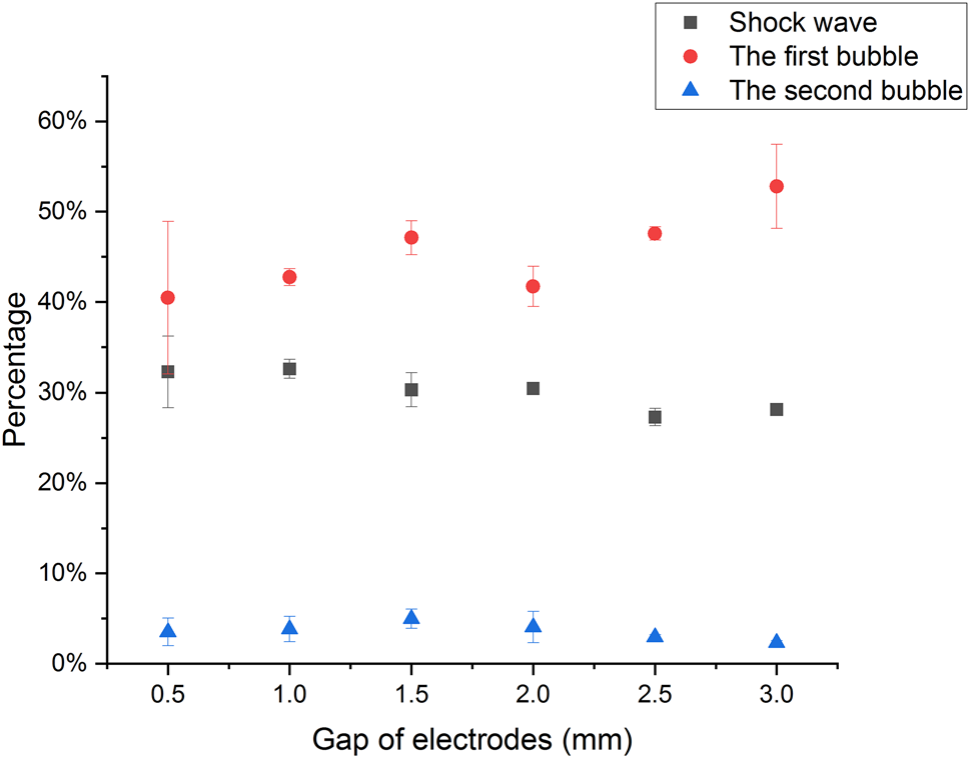
$E_{sh}/E$, $E_{FB}/E$, and $E_{SB}/E$ versus gaps.

With *E* standing for the total signal energy, the ratio of the energy of the shock wave to the one of the total signal is expressed as $E_{sh}/E$. Similarly, the ratio of the energy of the first bubble pulse to the one of the total signal is expressed as $E_{FB}/E$ and the ratio of the energy of the second bubble pulse to the one of the total signal is expressed as $E_{SB}/E$. These three quantities versus gaps are illustrated intuitively in Fig. 7. Compared to the first bubble or the shock wave, the second bubble pulse makes little contribution to the total energy of the acoustic signal. Therefore, the second bubble pulse is not our focus.

**Figure 8:**
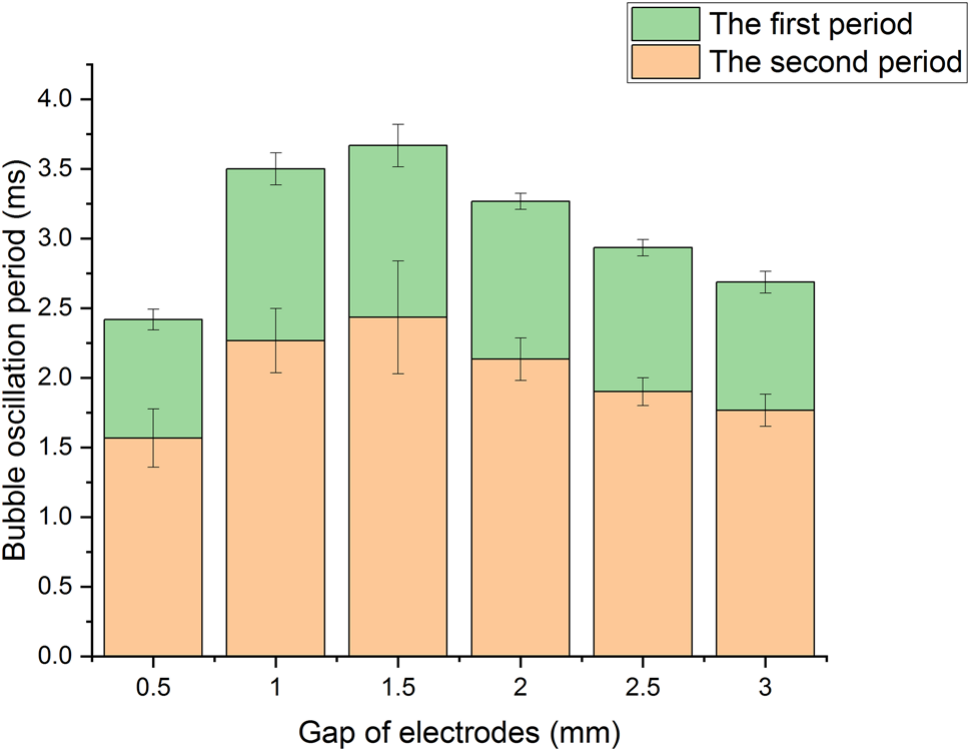
The bubble oscillation periods versus gaps.

As is shown in Fig. 8, the bubble oscillation periods compose of the first period which is the time interval between the peak time of the shock wave and that of the first bubble pulse, and the second period which is the time interval between the peak time of the first bubble pulse and that of the second bubble pulse. The first period, which is about twice the second period, has a range of 1.5 ms to 2.9 ms.

### Frequency-domain characteristics of acoustic signals produced by a spark discharge

Due to the high robustness to signal sampling and noise, VMD can suppress mode mixing to a certain extent. Therefore, VMD-HHT is our choice for analyzing the acoustic signal produced by underwater discharges. The key to VMD lies in constructing and solving variational problems. Constructing the variational problem takes four steps. Firstly, the original signal *f*(*t*) is decomposed into *K* IMFs, i.e., $f(t)=\sum \limits _{k=1}^{K}u_{k}(t)$, where $u_{k}(t)$ is the k-th IMF. $u_{k}(t)$ has an expression of $u_{k}(t)=A_{k}(t)cos(\phi _{k}(t))$, where $A_{k}(t)$ is the positive and slowly varying envelope and $\phi _{k}(t)$ stands for the phase. The instantaneous angular frequency of $u_{k}(t)$, which is non-decreasing, slowly varying and concentrating on a central value of $\omega _{k}$, is expressed as $\frac{\textrm{d}\phi _{k}(t)}{\textrm{d}t}$. Secondly, to ensure the positive spectrum of each IMF, the analytic signal of $u_{k}(t)$ is obtained as $(\delta _{t}+\frac{j}{\pi t})*u_{k}(t)$, where $\delta _{t}$ is the dirac delta function and $*$ is the symbol of convolution. Actually, the real part of the analytic signal is $u_{k}(t)$ itself and the imaginary part is the Hilbert Transform of $u_{k}(t)$. Thirdly, the analytic signal is multiplied by a factor of $e^{-j\omega _{k}(t)t}$ to modulate the spectrum of each IMF to the corresponding base frequency band. An expression is obtained as ${[}(\delta _{t}+\frac{j}{\pi t})*u_{k}(t)]e^{-j\omega _{k}(t)t}$, where $\omega _{k}$ is called the center frequency of $u_{k}(t)$. Lastly, the bandwidth is estimated as $\vert \frac{\textrm{d}}{\textrm{d}t}\left\{ [(\delta _{t}+\frac{j}{\pi t})*u_{k}(t)]e^{-j\omega _{k}(t)t}\right\} \vert _2^2$, where $\vert \cdot \vert _2$ stands for the 2-norm. The description of the variational problem is to minimize the sum of the estimated bandwidth of each IMF and the constraint condition is that the sum of all IMFs should equal to the original signal. Then the corresponding constraint variational expression is $min\sum \limits _{k=1}^{K}\vert \frac{\textrm{d}}{\textrm{d}t}\left\{ [(\delta _{t}+\frac{j}{\pi t})*u_{k}(t)]e^{-j\omega _{k}(t)t}\right\} \vert _2^2$, s.t.$\sum \limits _{k=1}^{K}u_{k}(t)=f(t)$. To get the solution, Lagrange Multiplication Operator $\lambda (t)$ is introduced to transform the constrained variational problem into the unconstrained variational one, and the augmented Lagrange expression is obtained as $L(u_{k},\omega _{k},\lambda )=\alpha \sum \limits _{k=1}^{K}\vert \frac{\textrm{d}}{\textrm{d}t}\left\{ [(\delta _{t}+\frac{j}{\pi t})*u_{k}(t)]e^{-j\omega _{k}(t)t}\right\} \vert _2^2+\vert f(t)-\sum \limits _{k=1}^{K}u_{k}(t) \vert _2^2+\left\langle \lambda ,f(t)-\sum \limits _{k=1}^{K}u_{k}(t) \right\rangle$, where $\alpha$ is the penalty factor measuring the importance of the first item on the right of the equal sign relative to the second and third items, $\langle \rangle$ stands for the inner product. Ultimately, the solution is obtained based on Eq. (1) and Eq. (2).

1 $$\begin{aligned} U_{k}^{n+1}(\omega )=\frac{F(\omega )-\sum \limits _{i=1,i<k}^{K}U_{i}^{n+1}(\omega )-\sum \limits _{i=1,i>k}^{K}U_{i}^{n}(\omega )+\Lambda ^n(\omega )/2}{1+2\alpha (\omega -\omega _{k}^n)^2} \end{aligned}$$

$U_{k}^{n+1}(\omega )$ is Fourier transform of the k-th IMF at the n + 1 Iteration, $F(\omega )$ is Fourier transform of the original signal *f*(*t*),and $\Lambda ^n(\omega )$ is Fourier transform of $\lambda$ at the *n* Iteration.

2 $$\begin{aligned} \omega _{k}^{n+1}=\frac{\int _{0}^{\infty } \omega |U_{k}^{n+1}(\omega ) |^2\, d\omega }{\int _{0}^{\infty } |U_{k}^{n+1}(\omega ) |^2\, d\omega } \end{aligned}$$

The Lagrange Operator is updated based on Eq. (3).

3 $$\begin{aligned} \Lambda ^{n+1}(\omega )=\Lambda ^{n}(\omega )+\gamma [F(\omega )-\sum \limits _{k=1}^{K}U_{k}^{n+1}(\omega )] \end{aligned}$$

$\gamma$ is the noise tolerance. The condition for stopping iteration is illustrated below.

4 $$\begin{aligned} \sum \limits _{k=1}^{K}\vert U_{k}^{n+1}(\omega )-U_{k}^{n}(\omega ) \vert _2^2/\vert U_{k}^{n}(\omega ) \vert _2^2<\varepsilon \end{aligned}$$

**Figure 9:**
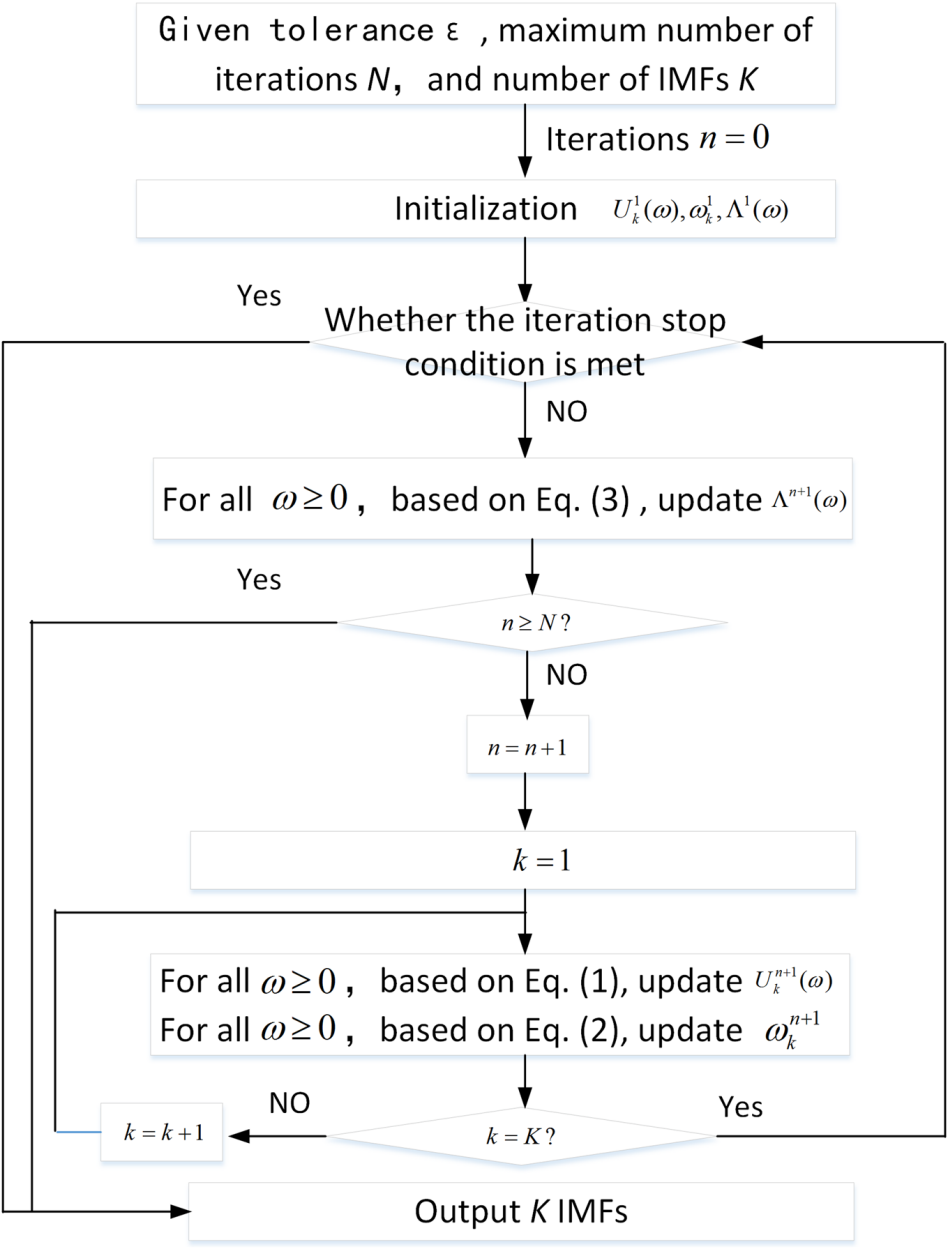
The flow chart of VMD.

The VMD algorithm can be illustrated as Fig. 9.

#### Determination of *K* value

Using VMD, the numbers of IMF, referred to as *K*, which are also called the decomposition numbers, have to be determined beforehand. If *K* is too small, the decomposition of the signal is insufficient and different frequencies will appear in the same IMF. If *K* is too large, the same or similar frequency will be decomposed into different IMFs, which will bring about an inadequate result. A common method to determine the *K* value is Central Frequency Method^25^. In the beginning, *K* is assigned a value of 2. If the central frequencies of IMFs differ greatly, then *K* is increased to 3. Repeat this progress until two IMFs have similar central frequencies when *K* equals to *k*. At this point, a conclusion is drawn that over-decomposition comes into being, and the optimal *K* is thought of as $k-1$. However, a problem to be solved is how to describe the similarity of the central frequencies. In most cases, the judgment is made manually based on experience. Unfortunately, this method does not go smoothly in processing our acoustic signal, with the sampling rate being as high as 50MHz. To facilitate subsequent signal processing, the sampling rate is dropped by 100 times but can not be furtherly reduced to a lower level due to the short duration of the signal. The minimum difference in different IMFs is several kHz even if *K* is increased to 20. We can not directly make a judgment whether there is over decomposition by the difference of central frequencies. Therefore, a relative center frequency difference method (RCFD) is proposed. The basic idea is simply clarified below. Firstly, the original signal is decomposed into *K* IMFs, where $K=2,3,\ldots ,k$, and we get a vector of center frequencies as $f_{cf}=[f_{c1},f_{c2},\ldots ,f_{cK}]$. Subsequently, a relative difference vector is expressed as $f_{rc}=[\frac{\vert f_{c1}-f_{c2}\vert }{f_{c1}},\frac{\vert f_{c2}-f_{c3}\vert }{f_{c2}},\ldots ,\frac{\vert f_{c,K-1}-f_{cK}\vert }{f_{c,K-1}}]$. Lastly, we calculate the difference between any two elements in this vector. If the minimum difference is less than one percent, similar central frequencies are thought to appear in $f_{cf}$ and over-decomposition occurs. Then, the optimal value of *K* is considered to be $k-1$. To verify the validity of RCFD, the energy difference method (ED) based on difference in root-mean-square energy of IMFs was used as a reference^26^. To do this, a vector is constructed as $\left\{ b_j\right\}$, where $b_j=\frac{\vert ET_{j+1}-ET_{j}\vert }{ET_{j}},j=1,2,\ldots ,k-1$, $ET_{j}=\sum _{i=1}^{j} \sqrt{\frac{\sum _{n=1}^{N} {c_{i,n}}^2(t)}{N}}$ with the data length of each IMF being *N* and the ith IMF being $c_{i,n}(t)$. $ET_1$ stands for the root-mean-square energy of the original signal. Therefore, the central frequency vector $\left\{ f_cf\right\}$, the relative center frequency difference vector $\left\{ f_rc\right\}$, and the root-mean-square energy difference vector $\left\{ b_j\right\}$ are calculated when $K=2,3,\ldots ,k$. Three methods to determine *K* value are compared, and the result is shown in Table 1.

**Table 1:** Three methods to determine *K* value.

$K=2$	Central frequencies (kHz)	[32.338,3.693]
	Vector of RCFD	0.8858
	Vector of ED	0.27
$K=3$	Central frequencies (kHz)	[52.244, 18.743, 2.502]
	Vector of RCFD	[0.6412, 0.8665]
	Vector of ED	[0.27 0.152]
$K=4$	Central frequencies (kHz)	[57.04, 33.766, 13.450, 2.009]
	Vector of RCFD	[0.408,0.6017,0.8506]
	Vector of ED	[0.27 0.152 0.126]
$K=5$	Central frequencies (kHz)	[83.394, 52.893, 32.435, 12.97, 1.953]
	Vector of RCFD	[0.3657,0.3868,0.6001,0.8494]
	Vector of ED	[0.27 0.152 0.126 0.0532]
$K=6$	Central frequencies (kHz)	[84.696, 53.738, 34.378, 18.241, 8.252, 1.436]
	Vector of RCFD	[0.3655,0.3603,0.4694,0.5476,0.826]
	Vector of ED	[0.27 0.152 0.126 0.0532 0.1012]

When $K=6$, the element of the central frequency vector is not similar. However, with RCFD used, the relative center frequency difference vector $f_{rc}=[0.3655,0.3603,0.4694,0.5476,0.826]$ and the minimum difference is 0.0052, which is less than one percent. Then over-decomposition is thought to occur, and the optimal *K* value is 5. The root-mean-square energy difference vector is obtained as $[b_1,b_2,b_3,b_4,b_5]=[0.27,0.152,0.126,0.0532,0.1012]$. We can see that $b_5$ is obviously greater than $b_4$. Therefore, the same result is obtained that the optimal value of *K* is 5. According to the analysis above, RCFD is an available method to determine the *K* value.

#### Analysis of the total acoustic signal

**Figure 10:**
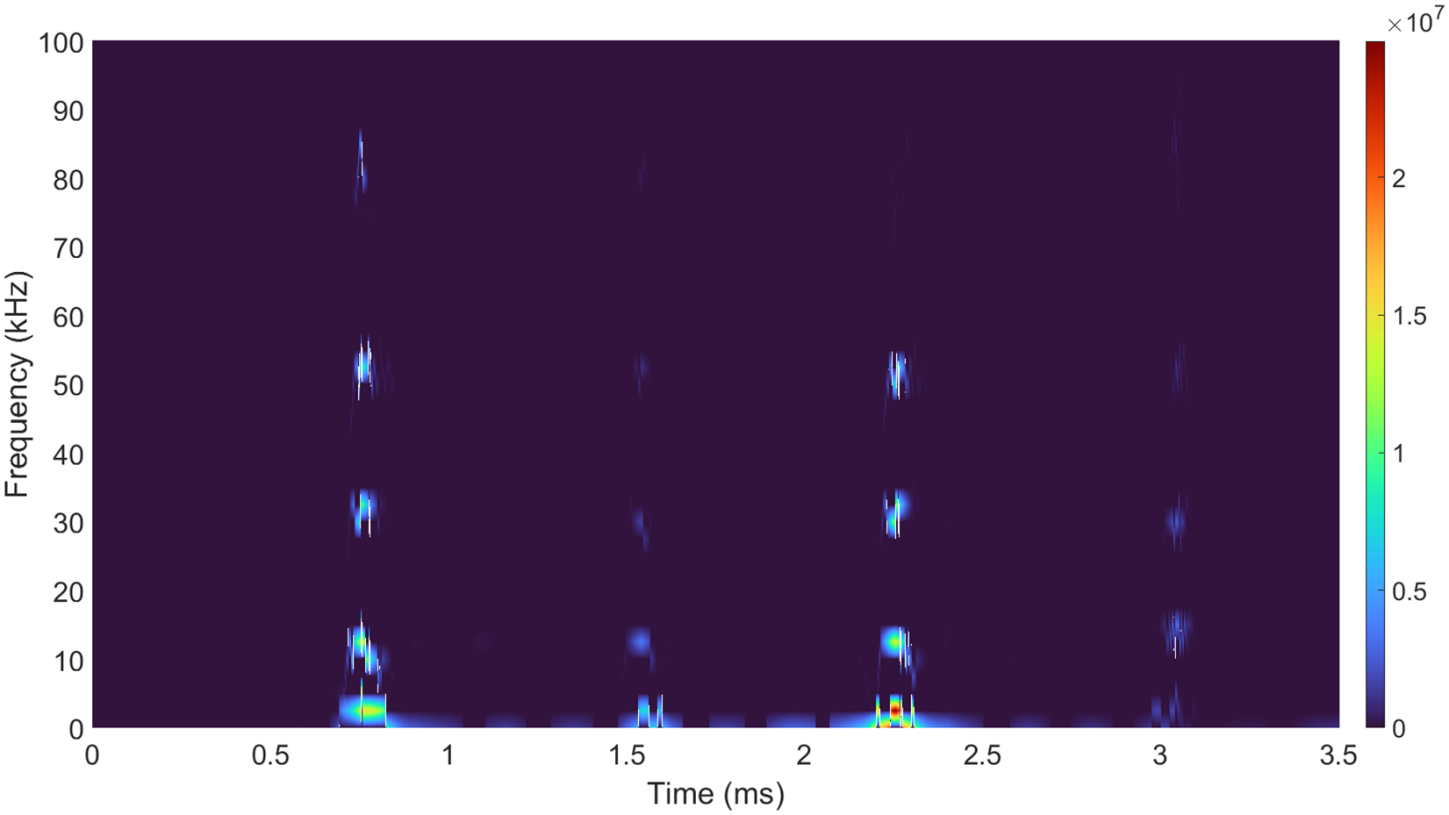
The Hilbert spectrum of the total acoustic signal.

The Hilbert spectrum of the total acoustic signal illustrated in Fig. 3 is computed and shown in Fig. 10.

**Figure 11:**
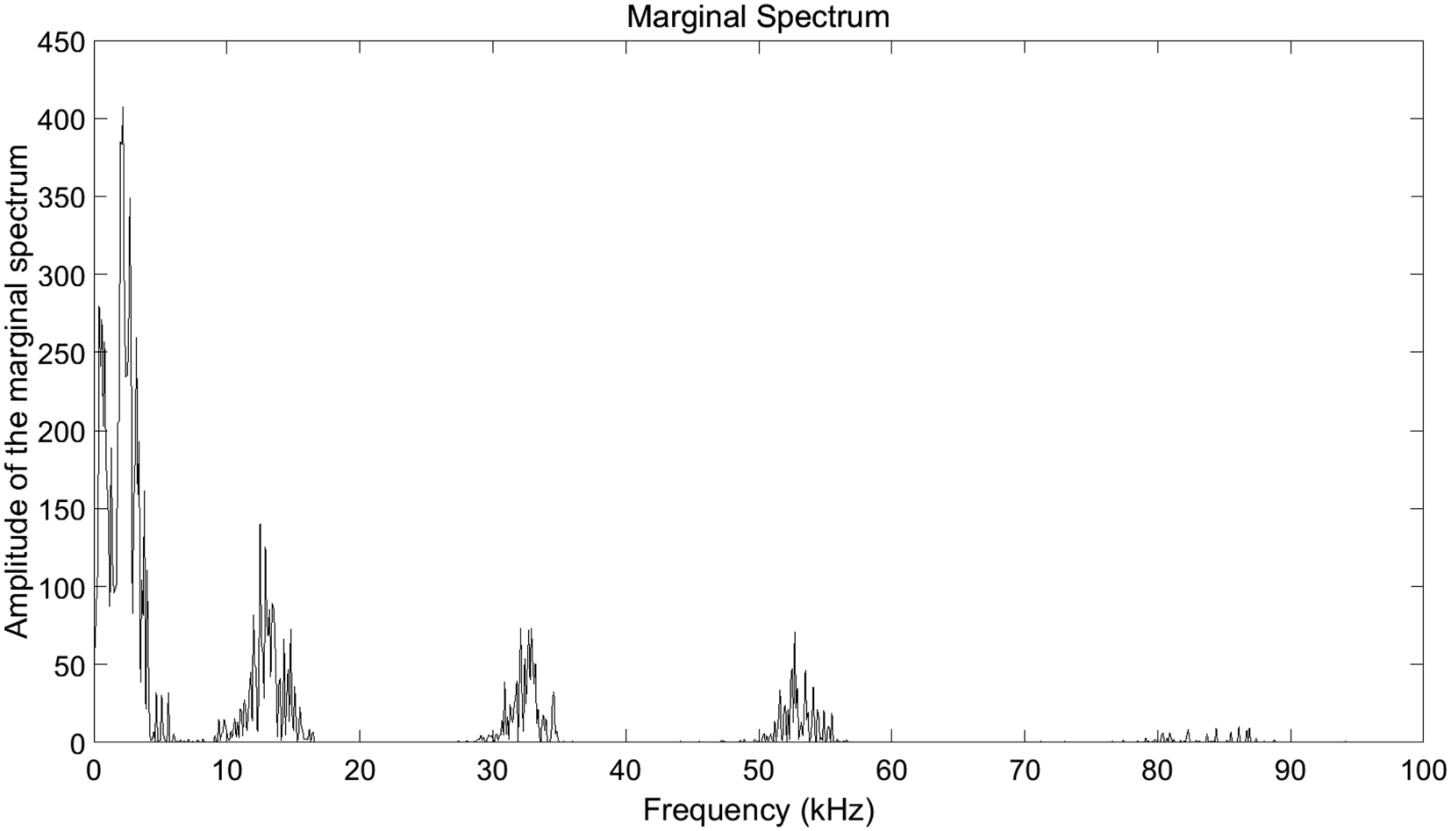
Marginal spectrum.

Both the frequency information and the moment the frequency appears are clearly demonstrated, which indicates that the time-frequency characteristics of the signal can be obtained by HHT. It can be seen intuitively that the shock wave contains more high-frequency components than the bubble pulse due to the more steep rising edge and the shorter pulse width. The color in Fig. 10 represents the strength of the signal energy. The radiated energy of the bubble pulse is obviously higher than the energy of the shock wave in the low-frequency band (0–4 kHz). Therefore, we get the knowledge that the high-frequency components of the signal produced by underwater discharges are mainly attributed to the shock wave, and the low-frequency components result from the bubble pulse mostly. The marginal spectrum^27^, which is shown in Fig. 11, is acquired through the time integral of the Hilbert spectrum and supplies more accurate frequency information than the FFT spectrum.

**Figure 12:**
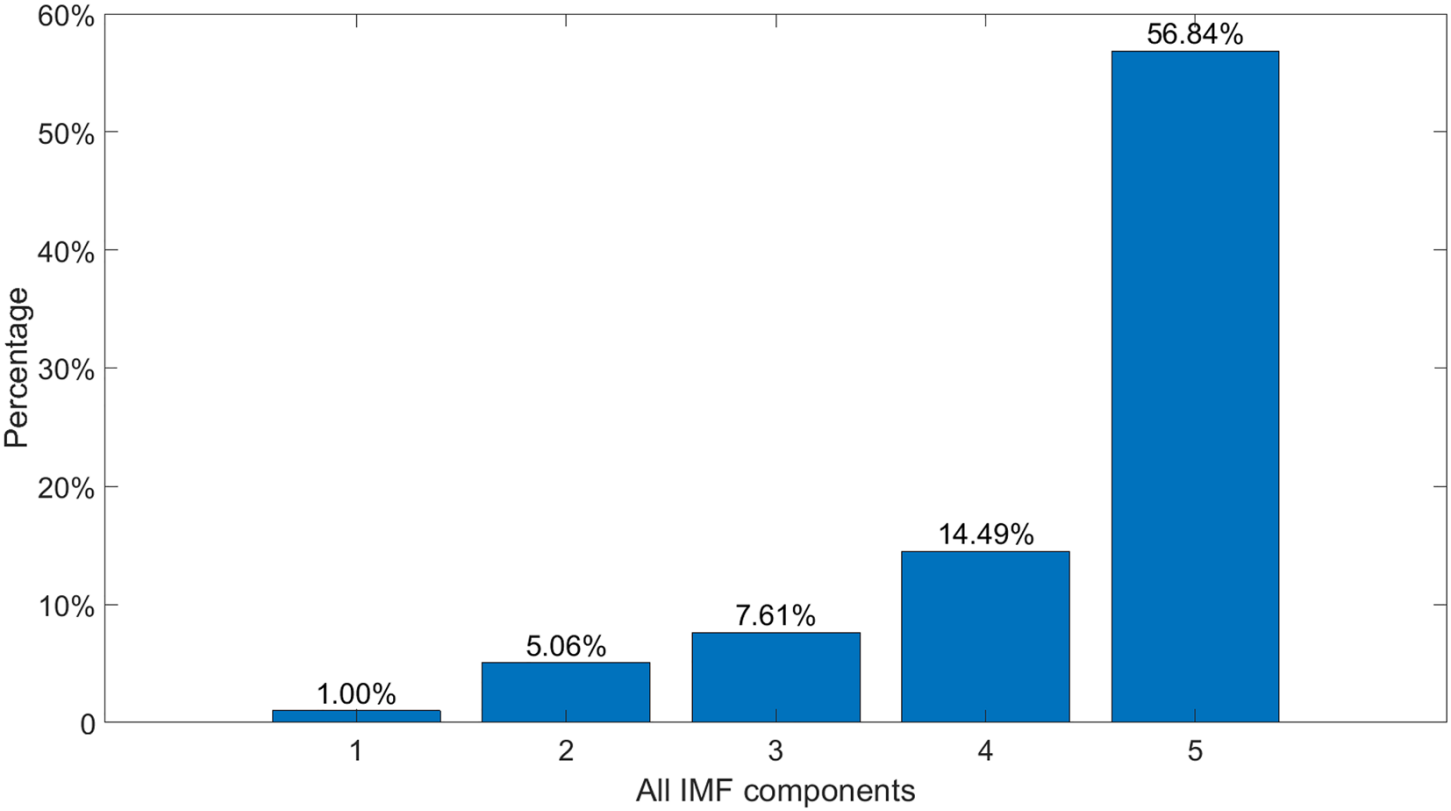
$$E_{imf}/E$$.

The wide frequency band of the acoustic signal has a range of 0–90 kHz, and the marginal spectrum of every IMF is clearly visual. IMF 1 has the smallest amplitude and the highest frequency, while IMF 5 is just the opposite. With $E_{imf}$ standing for the energy of an IMF, the percentage of $E_{imf}$ in *E* is shown in Fig. 12.

**Figure 13:**
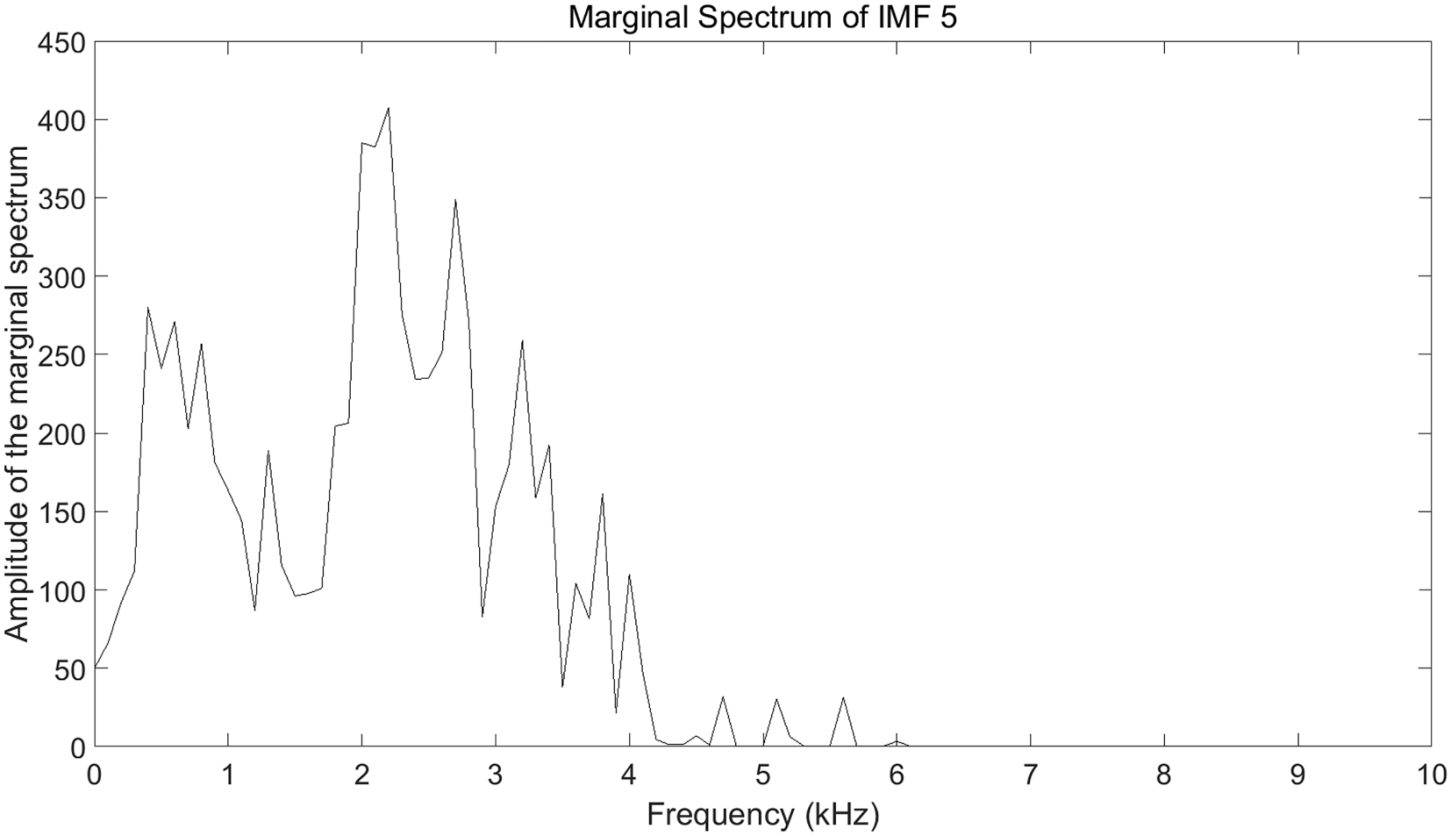
The marginal spectrum of IMF 5.

$E_{imf5}/E$ is as high as 56.84%, which draws our attention. Thus, the marginal spectrum of IMF 5 is plotted in Fig. 13 to illustrate the frequency characteristic clearly.

**Figure 14:**
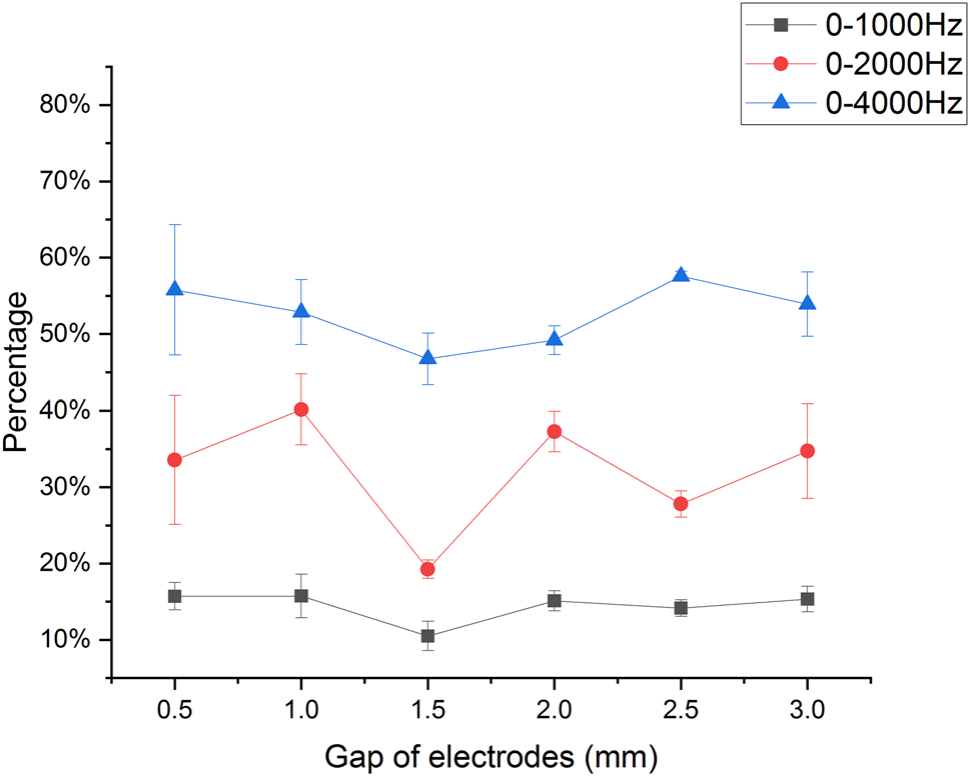
$$E_{\Delta f}/E$$ versus gaps.

IMF 5 ranges in frequency mainly from 0 to 6 kHz. The energy below 4 kHz accounts for 97.74% of the energy of IMF 5 and accounts for 55.56% of the total acoustic signal, which shows that the energy of the acoustic signal generated by underwater discharge is mainly concentrated in the low-frequency band. Meanwhile, at the frequency of 1 kHz and 2 kHz, the significant amplitude is obvious. Thus, our concerned frequency band is set as 0–1 kHz, 0–2 kHz, and 0–4 kHz. With $E_{\Delta f}$ standing for the energy in certain frequency band, the the ratio of $E_{\Delta f}$ to *E* versus gaps is shown in Fig. 14.

As is seen in Fig. 14, the changing trend of $E_{\Delta f}/E$ in all three frequency bands is just opposite to the one of the peak pressure and radiated energy. Specifically speaking, at the gap of 1.5 mm, which is the optimal gap for peak pressure and radiated energy of the acoustic signals, $E_{\Delta f}/E$ is the lowest. This may be caused by the violent oscillation of the acoustic signals due to the maximum deposition energy at the gap of 1.5 mm. The violent acoustic oscillation means more high frequency, which brings about the minimum of $E_{\Delta f}/E$.

## Conclusions

The time-frequency characteristics of the acoustic signals produced by underwater discharges are obtained by HHT in this paper. In consideration of suppressing mode fixing, VMD rather than EMD is chosen to decompose the signal. According to the principle of VMD algorithm, the decomposition numbers *K* of the acoustic signals has to be determined in advance. Therefore, we propose an relative central frequency difference method, namely, RCFD, and give one-percent judgment criteria to determine the *K* value. Then, The HHT spectrum is calculated and some useful conclusions are drawn. The high-frequency components of the signal are mainly attributed to the shock wave, and the low-frequency components result from the bubble pulse mostly. The acoustic signal has a frequency range from 0 to 90 kHz, and the energy in the low-frequency band (0–4kHz) is dominant in the total energy with a proportion of 55.56%. Also $E_{\Delta f}/E$ versus gaps is investigated. To get an accurate result, we calculate $E_{\Delta f}/E$ in 0–1 kHz, 0–2 kHz, and 0–4 kHz. The results show that $E_{\Delta f}/E$ gets the minimum at the gap of 1.5 mm and the changing trend is just opposite to the one of the peak pressure and radiated energy of the acoustic signal. In other words, we cannot get the maximum energy of the acoustic signal and the maximum $E_{\Delta f}/E$ in the three bands at the same time. This conclusion is helpful for the application of the acoustic signal produced by underwater discharges.

## Data Availability

Data available on request due to privacy/ethical restrictions:The data that support the findings of this study are available on request from the corresponding author. The data are not publicly available due to state restrictions.
